# Parametric Effects of Single Point Incremental Forming on Hardness of AA1100 Aluminium Alloy Sheets

**DOI:** 10.3390/ma14237263

**Published:** 2021-11-27

**Authors:** Sherwan Mohammed Najm, Imre Paniti, Tomasz Trzepieciński, Sami Ali Nama, Zsolt János Viharos, Adam Jacso

**Affiliations:** 1 Department of Manufacturing Science and Engineering, Budapest University of Technology and Economics, Műegyetem rkp 3, H-1111 Budapest, Hungary; imre.paniti@sztaki.hu (I.P.); jacso.adam@gpk.bme.hu (A.J.); 2 Kirkuk Technical Institute, Northern Technical University, Kirkuk 41001, Iraq; 3 Centre of Excellence in Production Informatics and Control, Institute for Computer Science and Control (SZTAKI), Kende u. 13-17, H-1111 Budapest, Hungary; viharos.zsolt@sztaki.hu; 4 Department of Manufacturing and Production Engineering, Faculty of Mechanical Engineering and Aeronautics, Rzeszow University of Technology, Al. Powst. Warszawy 8, 35-959 Rzeszów, Poland; tomtrz@prz.edu.pl; 5 Engineering Technical College, Middle Technical University, Baghdad 10074, Iraq; drsami@mtu.edu.iq; 6 Department of Management and Business Law, Faculty of Economics and Business, John von Neumann University, Izsáki Str. 10, H-6000 Kecskemét, Hungary

**Keywords:** SPIF, single point incremental forming, sheet forming, hardness, ANN, relative importance (RI)

## Abstract

When using a unique tool with different controlled path strategies in the absence of a punch and die, the local plastic deformation of a sheet is called Single Point Incremental Forming (SPIF). The lack of available knowledge regarding SPIF parameters and their effects on components has made the industry reluctant to embrace this technology. To make SPIF a significant industrial application and to convince the industry to use this technology, it is important to study mechanical properties and effective parameters prior to and after the forming process. Moreover, in order to produce a SPIF component with sufficient quality without defects, optimal process parameters should be selected. In this context, this paper offers insight into the effects of the forming tool diameter, coolant type, tool speed, and feed rates on the hardness of AA1100 aluminium alloy sheet material. Based on the research parameters, different regression equations were generated to calculate hardness. As opposed to the experimental approach, regression equations enable researchers to estimate hardness values relatively quickly and in a practicable way. The Relative Importance (RI) of SPIF parameters for expected hardness, determined with the partitioning weight method of an Artificial Neural Network (ANN), is also presented in the study. The analysis of the test results showed that hardness noticeably increased when tool speed increased. An increase in feed rate also led to an increase in hardness. In addition, the effects of various greases and coolant oil were studied using the same feed rates; when coolant oil was used, hardness increased, and when grease was applied, hardness decreased.

## 1. Introduction

Incremental Sheet Forming (ISF) is a sheet-forming technique that produces components through a series of small incremental deformations. ISF is a flexible active manufacturing process and is economically feasible for low-volume production due to the absence of a punch and die. SPIF is one of the major types of ISF, and it is known as the simplest process variant of incremental sheet-forming technologies. SPIF is an emerging process that has been identified as suitable for use in small-scale production. Trzepieciński et al. [1] presented a brief overview of state-of-the-art methods of ISF for lightweight materials. The aim of their paper was to guide and inspire researchers by identifying current development trends of valuable contributions in the field of Single Point Incremental Forming (SPIF) of lightweight metallic materials. In SPIF, a rotating tool with a rounded tip at its end is used, and the desired shape is formed from clamped sheet metal [2]. In the literature [3], a review paper on new advances and future opportunities considered single point incremental to be one of the forming technologies of future-proof materials in aerospace applications. Furthermore, there is governmental, academic, and business interest in developing new manufacturing technologies, and there is also interest in ISF’s impact on the environment, particularly in what ways and to what extent ISF reduces energy needs [4]. Two exergy analyses of traditional forming and hydroforming of ISF were contrasted by Dittrich et al. in [5]. After analysing the environmental impact of these forming techniques in the supply chain, they concluded that ISF is significantly less harmful to the environment, particularly for prototypes and non-mass production. Sustainability guidelines were developed by Ingarao et al. [6] regarding the advantages and disadvantages of SPIF related to the amount of energy necessary to form sheets and to economic material use in each process. Ingarao et al. proved that SPIF supports saving material with respect to CO_2_ emissions, because it supports recycling and facilitates novel ways of preparing raw materials. An overview of the history of ISF was written by Emmens et al. [7], Li et al. [8], and Behera et al. [9]: they discussed the enormous benefits and many advantages of SPIF and particularly referenced the flexibility of the process, which allows SPIF to be used in more applications in industries and processes. Hence, SPIF will be considered an essential process for the industry in the future.

By studying the mechanical properties of components formed through SPIF and standardising effective process parameters, the SPIF process can become a significant industrial application embraced by numerous companies. To set optimal input process parameters of SPIF with multiple pure copper sheets, Raju and Sathiya [10] utilised a hybrid optimisation technique by connecting it with Taguchi grey relational analysis and the methodology of the response surface. They showed that the feed rate is the most significant parameter, followed by step depth and tooltip diameter. On the other hand, lubrication plays a vital role in the successful forming of components during the SPIF process. Lubrication reduces friction at the contact zone of the tool and sheet [11]. Recently, José et al. [12] studied how mineral oil, sunflower, soybean, and corn lubricants influence friction and wear effects on aluminium parts manufactured using the SPIF process. Using a scanning electron microscope, they found the following: the experimental characterisation of the sample’s surface showed that the worn surface of the metallic material samples produced using vegetable oils increases surface roughness compared to those produced with mineral oils. With respect to aluminium alloy foils, Najm and Paniti [13,14] noted that a flat tool yielded better outcomes than a hemispherical tool in various conditions of the SPIF process. The best geometric accuracy was achieved when the smallest corner radius flat tool was used because a decrease in spring-back was observed. ISF-as-a-Service was introduced by Paniti [15], who distinguished first-order and second-order bottleneck parameters. He described the main capabilities of an incremental sheet-forming service provider in cloud manufacturing. The hardening and normal anisotropy coefficients are the most influential factors on traditional Forming Limit Diagrams (FLDs), as found by Fratini et al. [16] when studying the relationship between material formability and mechanical properties. On the other hand, Zhang [17] concluded that the most influential factor on formability is forming temperature, followed by vertical step depth, sheet thickness, and tooltip diameter. The above-mentioned results were obtained on Mg alloy sheets formed by using warm incremental sheet forming. Liu et al. [18] asserted that formability and maximum vertical force increase when vertical step-down increases, and they linearly increase when sheet thickness is increased. In their study [19], Li et al. found that mechanical properties and the thinning rate were affected by three parameters of ISF. It was shown that an increase in the tool diameter considerably improved the microhardness of the product’s surface. An increase in the tool diameter and a decrease in the step size ultimately increase the tensile strength rate. Li et al. claimed that contrary to step size, sheet thickness significantly affects yield strength. In [20], Manco et al. proved that, due to variations in tool path, tool trajectory could be considered an essential parameter for the optimisation of process design by comparing the smallest thickness of the sheet with predicted thickness using the sine law. Krasowski et al. [21] analysed and discussed experimental investigations of the effects of selected SPIF parameters on the formability of DC04 sheets and the susceptibility to crack formation on truncated cones produced through SPIF, and they found that lubrication conditions clearly affect the formability of DC04 steel sheets.

At present, many methods of artificial intelligence are used in various applications, including the metal forming process. More specifically, ANN generates predictive models for end-milling machining, powder metallurgy, and high-speed machining [22,23,24]. Moreover, machine learning techniques with controlled manufacturing are used to develop various effective predictive models [25,26,27,28,29]. Trzepieciński et al. [30] presented an analysis of the interaction between SPIF process parameters and the main roughness parameters. They found that predictive models of ANNs for Ra and Rz were characterised by performance measures of R^2^ values between 0.657 and 0.979. In other studies, different tool materials and shapes were investigated experimentally to study factors including formability, geometric accuracy [31], and surface roughness [32] on an AlMn1Mg1 sheet formed using SPIF under various forming conditions. The researchers evaluated the performance of an Artificial Neural Network (ANN) and Support Vector Regression (SVR). Two different ANN models were built in the study: an R-squared value with other validation metrics and a feed-forward neural network with a backpropagation algorithm were used. A close correspondence was found between predicted roughness, formability, and geometric accuracy in the experimental results. The researchers derived regression equations to analytically predict surface roughness in terms of Ra and Rz. Baruah et al. [33] claimed that lubrication was the largest contributing factor in the process of ISF in all three directions (rolling, transverse, and angular) when surface roughness in ISF is meant to be reduced. In fact, to date, the applied lubricant and the viscosity of the lubricants on the ISF process have not been optimised or discussed, as attested by [5,34,35]. In addition, Kumar and Gulati [34] claimed that all parameters investigated in their study were significant for forming force except lubricating oil viscosity, and they also noted that surface roughness decreased when viscosity increased [35]. According to the literature, ANN is a helpful tool—before starting new experiments—for predicting and designing predictive models to estimate expected results, behaviour, or direction based on the use of the parameters of the studied process. Using ANN before starting an actual experiment has the essential benefits of selecting the correct parameters, reducing processing time, increasing efficiency, minimising errors, and comparing actual results with predicted ones so as to reach the best values. In addition, ANN is considered one of the most powerful tools for solving engineering problems by predicting experimental data. In addition, ANN can serve as a valuable means to generate and assess different processes and prepare the final details of tools.

Under normal conditions, the hardness behaviour in SPIF is as follows: formed parts achieve higher hardness than an unformed sheet. Using different path strategies and different forming angles, Al-Attaby et al. [36] showed that the tool path affects the hardness and microstructure of the formed sheet. In all cases relating to the forming angle, hardness increased. Regarding the two-point incremental forming process, Mostafanezhad et al. [37] analysed the formability of aluminium 1050: the scholars used the response surface methodology experimentally. They found that wall angle is the most influential factor with respect to the thinning ratio; initial thickness, followed by step-down, has a significant impact on forming force. 

The above-detailed issues, the need for well-defined mechanical properties of SPIF components, and the lack of referent analytical models prompted the authors to investigate the effects of SPIF process variables on the hardness of truncated cones formed from AA1100 aluminium alloy sheets. Moreover, as a novelty and aim contributing to the significance of this paper, different regression equations were derived to determine the hardness of the components of a truncated cone using SPIF. In addition, the Relative Importance (RI) of parameters of SPIF on hardness was assessed and classified by utilising the joint partitioning weight of the built neural network. To the best of the authors’ knowledge and according to the literature introduced, such an experimental process has not been reported to date. In this research, the influences of feed rate, various kinds of grease and coolant oil, spindle speed, and tool diameter on the hardness of the AA1100 aluminium alloy sheet formed by SPIF were investigated. Conventionally, AA1100 alloy is employed for radiator components [38]. However, as a final general aim, it was posited that the appropriate selection of properties would improve the application of the AA1100 alloy. Furthermore, based on the diver’s mechanism of deformation, Song et al. [39] found three different regions of deformation (bending/stretching, shear, and stretch/shear). Based on these research projects, in this study, hardness was measured in three different positions on the inside wall of the cone, and measured data were compared to the primary hardness of the sheet involved in the experiment.

## 2. Material and Methods

### 2.1. Workpiece Material

Aluminium and aluminium alloys have become attractive materials for application in the aerospace and automotive industries owing to their beneficial properties. In the experiments conducted in this study, single point incremental forming tests were conducted using a blank sheet of AA1100 aluminium alloy were produced by Xuzhou Bozhan Aluminum Technology Co. Ltd, Xuzhou, Jiangsu, China with an initial hardness of 42.87 HV. The initial thickness of the sheet used is 0.6 mm, with an original surface roughness of 0.29µm. AA1100 aluminium belongs to the 1xxx series with less than 1% alloying elements. The main uses of the 1xxx series aluminium alloys are foil and strip for packaging, chemical equipment, tank car or truck bodies, spun hollowware, and elaborate sheet metal work because of their high corrosion resistance and formability [40]. The 1xxx alloys are essentially characterised by superior corrosion resistance, usefulness for fabricating chemical tanks and piping, or their excellent electrical conductivity, as in bus bar applications. These alloys have relatively poor mechanical properties [41].

If strength is not an essential factor, AA1100 aluminium is selected to create fuel tanks, cowlings, and oil tanks of aircraft due to the corrosion resistance and the economic weight. The before-mentioned grade of aluminium can be utilised to repair aircraft wingtips and tanks because it is weldable [42]. AA1100 commercially pure aluminium is highly resistant to chemical attack and weathering. This low-cost material is characterised by excellent solderability and susceptibility to deep drawing. It is used for high-purity applications such as chemical processing equipment. In addition, examples of common 1xxx series aluminium alloy applications include nameplates, fan blades, flue lining, sheet metal work, spun holloware, and fin stock [43]. It is also used to produce decorative parts, giftware, cooking utensils, rivets, and reflectors. A SPECTROMAXx optical emission spectrometer manufactured by SPECTRO, Kleve, Germany was used to determine the chemical composition of the AA1100 alloy used, and the test of mechanical properties was conducted with a United testing machine according to the ASTM B557M-15 standard test methods for tension check. The results of the tests of the aluminium sheet were compared with the nominal values in the ASM Handbook [44]: these tests show the conformity to the standard composition of the alloy (ISO 19000 standard). The mechanical properties and the chemical composition of the sheet material are shown in Table 1 and Table 2, respectively.

### 2.2. Experimental Setup

**Figure 1:**
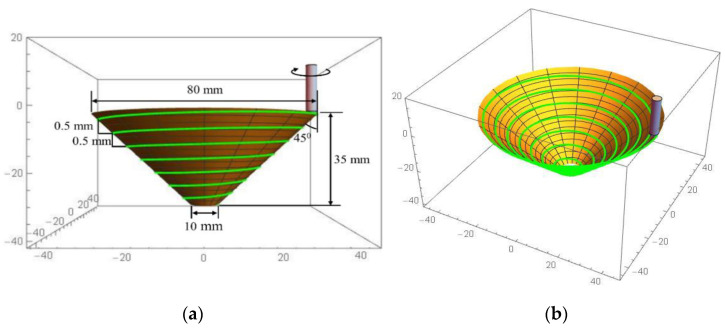
(**a**) CAD geometry and dimensions of the experimental product and (**b**) view of an inward spiral path.

In this study, a Boxford 300VMCi milling machine built by Boxford in Halifax, UK with 0.01 mm accuracy was used. The ISO format using G and M codes was used to program a cone shape with large and small diameters of 80 and 10 mm, respectively. An inward spiral path strategy was used to deform the cone part, in which case a spiral tool trajectory is advantageous to the successful forming of the same parts [45]. The strategy utilised in this study was developed by Skjoedt et al. [46] to overcome the difficulty of reaching maximum axial loads at each layer (step down) and to prevent the appearance of a line on the inner side of the formed part. A wall angle of 45°, a context contour of 0.5 mm for the step size, and an inward spiral path were applied, as shown in Figure 1.

In the SPIF process, only one tool can be used, and the feed rate, spindle speed, lubricant, and forming conditions should be selected beforehand. Furthermore, in the current study, the mentioned parameters were considered parametric values and were changed in the subsequent part forming. A primary step was conducted to select the best values of these parameters to fix them in the subsequent forming process, and only one of these parameters was changed in each forming group. To this end, a matrix of 3 factors with the same levels (feed rate, spindle speed, and tool diameter) was applied in the first step. The best values of feed rate, spindle speed, and tool diameter were chosen depending on the best geometrical accuracy and maximum depth. In the second step, the best values selected from the first step were applied in order to study the coolant type.

**Figure 2:**
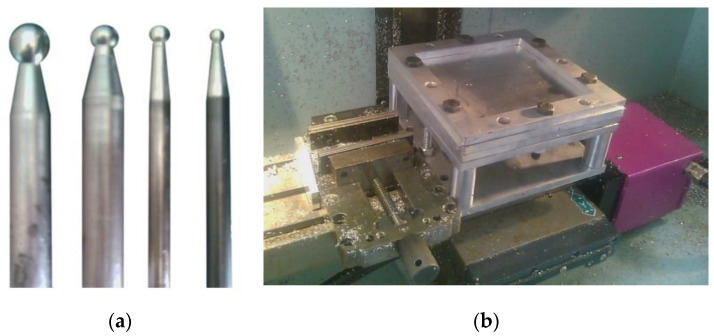
(**a**) Forming tools with different diameters, (**b**) fixture rig on the CNC machine table.

Forming tools with different diameters (4, 6, 8, and 10 mm) were used in the experiment, as shown in Figure 2a. The tools used in the experiment are made of carbon steel with a hardness of 30 HRC and are 100 mm in total length. Plain carbon steel was used for manufacturing the clamping rig, which was fixed to the CNC machine table with a simple-to-use fixture system, as shown in Figure 2b.

**Figure 3:**
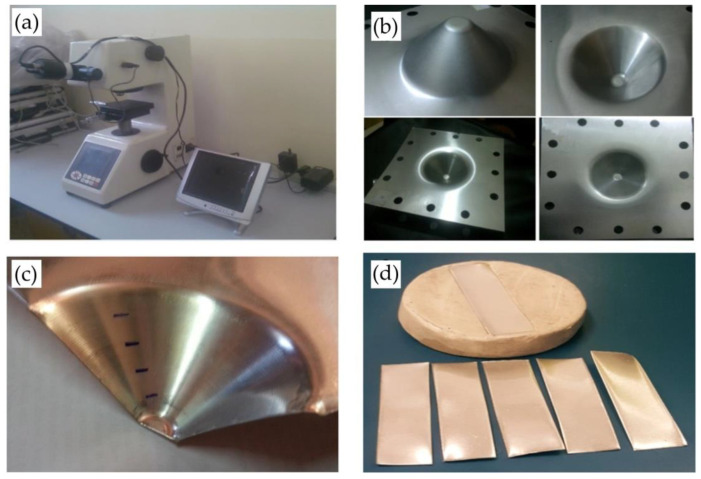
(**a**) Digital microhardness device, (**b**) formed parts, (**c**) three zones of hardness measurement, (**d**) hardness test sample of the formed part.

A digital Vickers microhardness tester supplied by TIME Group Inc., Beijing, China was used to measure the hardness of the component formed using SPIF (see Figure 3a) based on Equation (1). For each set, three products were experimentally formed to study the process parameters of different forming conditions (see Figure 3b). The hardness of each part was measured at three zones: the upper, middle, and lower zones along the inner wall of the formed part. Figure 3c shows a formed part after it was cut to the desired shape in the proper size for the preparation of the test samples and for establishing the three zones of the hardness measurement. The hardness measurement was repeated three times at different points inside each mentioned zone. The hardness value of each zone was calculated as the average value of the selected zone hardness. The average hardness value of the three zones was considered the average value of the measured component. The appropriate piece of the section was mounted by a mounting press device and polished by a Metaserv type 250/RP device manufactured in (Buehler, Lake Bluff, IL, USA) before hardness was measured (see Figure 3d).

(1) $$H_{v}=1854.4\;\frac{F}{d^{2}}$$

where *F* is penetration force (N), and *d* is average diagonal distance (d1 + d2)/2.

For hardness measurement, the hardness tester was calibrated before testing using the calibration standard block, and 100 N was applied on the formed part with a Vickers diamond pyramid indenter for 15 s. The results were recorded automatically on a digital screen after the adjustment of the rhomb corner had been triggered by the indenter.

**Figure 4:**
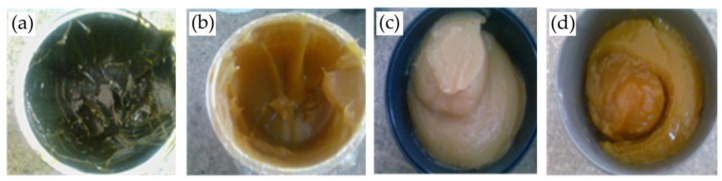
Different types of grease: (**a**) EP2, (**b**) Kaucuklu, (**c**) Zinol, (**d**) Gp Grease Calcium.

There is no internationally accepted term for the definition of Environmentally Acceptable Lubricants (EALs), and they still lack standardisation. The American Society for Testing and Materials (ASTM) used “environmentally acceptable” as a phrase for defining EALs [47]. There is an overall trend towards using EALs. In the present study, different coolant types (four different grease types, as shown in Figure 4a–d, and one coolant oil) were used to carry out the experiment. Supergrees EP2 and Kaucuklu grease produced by Petrol Ofisi, Istanbul, Turkey, Zinol grease from Universal Lubricants (ZINOL) L.L.C, Sharjah, United Arab Emirates, Gp Grease Calcium type was produced by United Grease & Lubricants Co. LLC based in Ajman, United Arab Emirates, and the coolant oil was also by Petrol Ofisi, Istanbul, Turkey. Table 3 lists grease properties based on their commercial name and standard denominations, and Table 4 presents coolant oil properties. It is worth mentioning that viscosity values of different greases were assumed based on the ISO 3448:1992 standard for viscosity grading systems [48].

Lubricants cannot be used in forming processes where high loads are applied, and thus, Syahrullail et al. [49] suggested using an appropriate additive to solve this problem. Consequently, the difference between using coolant oil and grease is that grease forms a mixture with small disintegrating particles (debris) of either the formed sheet or, in rare cases, the tool. Due to heat generation, sometimes the debris repeatedly sticks to the sheet surface or passes between the tool and the formed sheet. Diabb et al. [50] observed aluminium flakes in the used lubricant: this phenomenon was caused by wear adhesion on alloy sheets of SPIF components. In the case of coolant oil, which flows continuously on the sheet, debris can be washed away from the forming zone. However, when grease is used, a smoother surface can be produced compared to the scenario where coolant oil is used due to the flattening and roughening effects exerted by the debris, as stated in [51].

**Figure 5:**
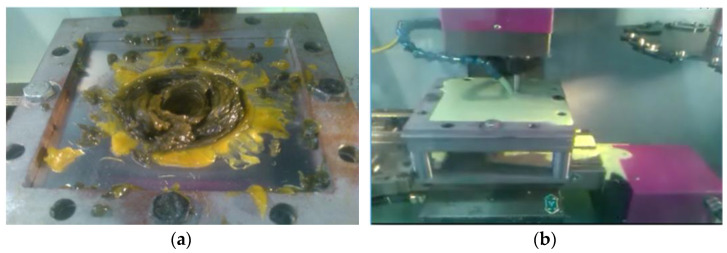
(**a**) Forming process using grease, (**b**) forming process using coolant oil.

On the other hand, coolant oil continuously flows during the forming process, whereas grease is applied on the sheet surface only once at the beginning of the process. In other words, coolant oil has higher exergy than grease due to the difference in the amount of material used, which means that an increased environmental impact is observable. Figure 5a,b illustrate the processes of using grease and coolant oil.

Four different tool rotation speeds (500, 1000, 1500, and 2000 rpm) were used to study hardness behaviour. In addition, in the scope of the current experiments, four different feed rates (200, 400, 600, and 800 mm/min) were implemented to investigate the effects of changes in the feed rate on sheet hardness.

## 3. Results and Discussion

### 3.1. Feed Rate

**Figure 6:**
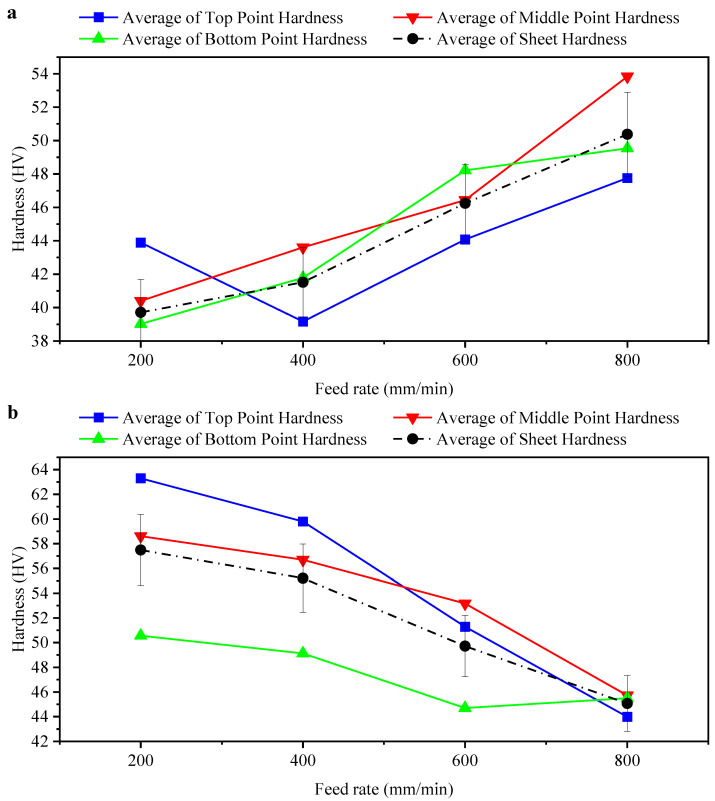
(**a**) Effects of different feed rates on hardness of formed sheet when using coolant oil, (**b**) effects of varied feed rates on hardness of formed sheet when using grease.

Four different feed rates were used with different lubricants (oil and grease). At the same time, other experimental parameters were fixed: the tool speed was 2000 rpm, the tool diameter was 10 mm, and coolant oil was used. Table 5 and Figure 6a,b show the results of hardness measurements for different feed rates. Changing the lubricant type resulted in inverse values of hardness: it increased when the feed rate was increased and coolant oil was used, and it decreased when grease was used.

An increase in feed rate led to an increase in hardness, and this was inversely proportional to formability. The increase in feed rate caused a decrease in formability, as mentioned in [52]. A decrease in hardness is due to changes in surface asperities because the peaks of the aspirates formed by the generated debris shoot and break. By attaching the debris to the tool and cultivating the sheet surface, new grooves can be created, and the sharp peaks of the asperities can likewise be crashed. Finally, through continuous cultivation and crashing, the contact area between the tool and the formed sheet will increase.

Hol et al. [53] mentioned that, in the case of normal forces, the sheet surface asperities are in the plastic condition, and they are further affected by only a little stress in the underlying bulk material. They claim that this stress is perpendicular to the normal force and generates increased plastic deformation of asperities. Finally, because of the enormous strain of the underlying material, this situation leads to an increased contact area, which is recognised as a decrease in effective hardness.

### 3.2. Tool Speed

**Figure 7:**
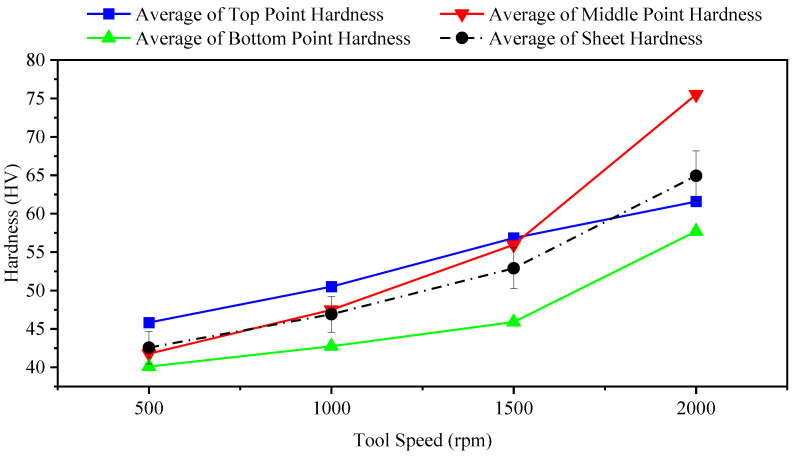
Effect of different tool speeds on hardness of formed sheet (feed rate: 600 mm/min; tool diameter: 10 mm; coolant: oil).

Table 6 lists different tool rotation speeds with the experimentally obtained hardness values. Figure 7 shows that an increase in tool speed led to an increase in hardness. High speed causes the resulting particles to impact the surface of the sheet faster than in the case of low speeds, and this results in the hardening of the surface. On the other hand, in the case of high tool speeds, the tool head, in the same contact area, travels on the sheet with more passes than a tool at low speeds. Due to stretching with longitudinal deformation, the sheet material seems to be undergoing cold working conditions. Cold working creates a different type of crystal deformation, such as compressing, twisting, and bending, and this results in comparatively uniform plain crystalline particles. New imperfections created by these movements result in more resistance and, finally, increase hardness.

### 3.3. Tool Diameter

**Figure 8:**
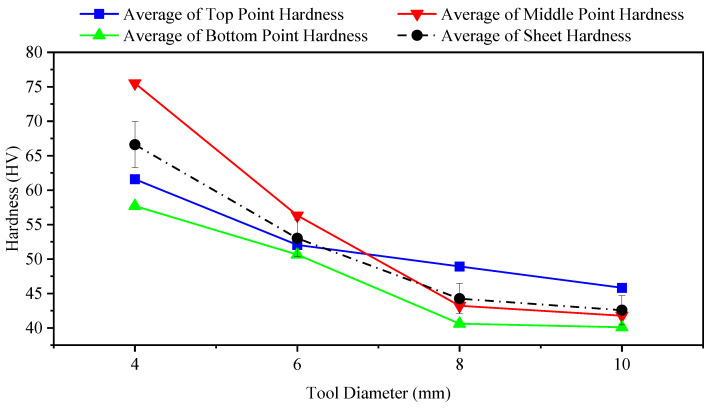
Effect of different tool diameters on hardness of formed sheet (feed rate: 600 mm/min; tool speed: 2000 rpm; coolant: oil).

The effects of tool diameter on hardness are presented in Table 7 and in Figure 8. Decreases in values are due to increases in tool diameter. McAnulty et al. [54] found different behaviours for the effects of changes in tool diameter on formability. Asgari et al. [55] concluded that a tool diameter of 3 mm results in increased hardness in an aluminium alloy 1100-O sheet relative to 5 or 10 mm tool diameters. A decrease in tool diameter from 10 to 3 mm causes ultimate tensile stress and yield stress to decrease by 7% and 24%, respectively. Furthermore, a reduction in the tool diameter causes a decrease in grain size [55]. Shrivastava and Tandon [56] discussed various parameters of the pre-production sheet and studied the effects of such parameters on the ISF process and on the final properties of products. They claimed that the forces needed to form the sheet in ISF are affected by grain size. Increasing the grain size leads to a decrease in forming forces, yield stress, and hardness [56]. The researchers used different tools for forming, and all of the formed parts showed decreased hardness irrespective of the diameter of the applied tools. The results of this study show that hardness decreased in the case of any diameter of the tool irrespective of the hardness value at each point. A tool diameter of 4 mm showed higher hardness than other diameters at all points. Tool diameters of 10 and 8 mm, on the other hand, produced lower hardness values than tool diameters of 6 and 4 mm. In addition, larger tools, which passed through the formed sheet more times than smaller tools, made the material of the formed part softer (and also caused more heating through increased friction).

### 3.4. Grease Grade

**Figure 9:**
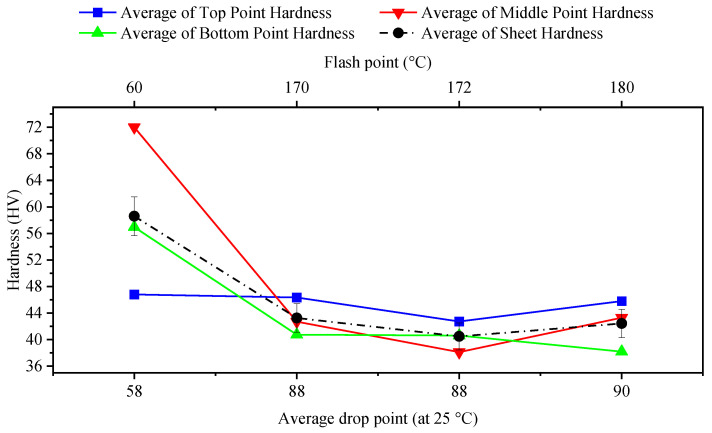
Effects of different grease types on hardness of formed sheet (feed rate: 600 mm/min; tool speed: 2000 rpm; tool diameter: 10 mm).

Table 8 and Figure 9 show the impact of different greases on the hardness results. The grease with the highest drop point resulted in the lowest hardness and vice versa. The grease type named “Gp Grease Calcium-ISO VG 46” provided the highest hardness (with the lowest drop point): this was due to the fact that this grease entered between the tool and the formed sheet and cooled the local forming zone faster. Regarding the properties of different greases, it is shown that the use of the grease with a higher flash point resulted in a more stable hardness value. In fact, it can be noted that the use of grease rather than the use of coolant oil produced more homogeneous hardness values at different points of the same sheet, with a slight difference in this conclusion for coolant oil compared to Gp Grease Calcium type grease.

### 3.5. Regression Equations to Calculate the Hardness of SPIF Components

Regression enables one to find an alternative method to quickly and more economically calculate SPIF components’ hardness, rather than having to resort to an experimental process. In view of this, in our experiments, regression equations capable of calculating the hardness of SPIF were used instead of actual measurements. Consequently, different equations were used in this study: Equation (2) Linear Cross-Validation Regression, (3) Linear Cross-Validation with Multiple Regression of Viscosity, (4) Multiple Regression, and (5) Equation Based on Biases and Weight. These equations are as follows:

Linear Cross-Validation Regression:

(2) $$H=\left(Fr_{Coeff}\times Fr+S_{Coeff}\times S+D_{Coeff}\times D+L_{Coeff}\times L_{v}+C\right)/4\;H=\left(0.0658\times Fr+0.0028\times S-9.3993\times D+0.2829\times L_{v}+234.7775\right)/4$$

Linear Cross-Validation with Multiple Regression of Viscosity:

(3) $$H=\left(Fr_{Coeff}\times Fr+S_{Coeff}\times S+D_{Coeff}\times D+(L_{Coeff1}\times L_{v}-L_{Coeff2}\times L_{v}{}^{2}\right)+C)/4\;H=\left(0.0749\times Fr+0.007482\times S-8.2277\times D+\left(3.5691\times L_{v}-0.0950\times L_{v}{}^{2}\right)+217.7624\right)/4$$

Multiple Regression:

(4) $$\begin{array}{l} H=(Fr_{Coeff1}\times Fr-Fr_{Coeff2}\times Fr^{2})+(S_{Coeff1}\times S-S_{Coeff2}\times S^{2})\\ \;+(D_{Coeff1}\times D^{2}-D_{Coeff2}\times D)+(L_{Coeff1}\times L_{v}{}^{2}\\ \;-L_{Coeff2}\times L_{v})+C\\ H=(0.029255\times Fr-0.000010\times Fr^{2})+(0.0278\times S-0.000009\times S^{2})\\ \;-(17.4122\times D-1.0414\times D^{2})\\ \;-(0.9343\times L_{v}-0.0244\times L_{v}{}^{2})+87.4763 \end{array}$$

Equation Based on Biases and Weights:

(5) $$H=b_{2}+LW\times Exp^{\left(-{\left(b_{1}+IW\times x\right)}^{2}\right)}\;\begin{array}{cc} H=3.9206+\; & \left[LW\right]\times exp^{\left(-{\left(\left[\begin{array}{c} -5.1413\\ 1.2616\\ 0.6642\\ 2.7010\\ 1.5268\\ -2.5527\\ 0.9677\\ 0.9160\\ 2.3191\\ -4.4413 \end{array}\right]+\left[\begin{array}{cccccccccc} -1.1773 & -0.7032 & -1.7944 & -1.8489 & 1.2188 & 2.0108 & -0.6296 & 1.9369 & 0.7717 & 0.3779\\ -1.7346 & -1.1106 & 0.1240 & 0.7292 & 0.0289 & -0.3600 & 0.6209 & -2.3675 & -0.6774 & -1.6394\\ 0.3222 & -0.2106 & -0.1155 & 1.1132 & 2.3911 & -0.5010 & -1.7080 & 0.0188 & 0.4250 & -0.6822\\ 0.1749 & -0.6833 & -0.8397 & 1.7357 & -1.2086 & -0.6854 & -0.8242 & 1.5373 & -1.4357 & 0.2100 \end{array}\right]\;\times \;\left[\begin{array}{c} x\\ Fr\\ S\\ D\\ L \end{array}\right]\right)}^{2}\right)} \end{array}$$

where $\begin{array}{cccccccccc} LW=[6.4552 & -2.1276 & 2.7984 & 7.8625 & 4.7908 & 4.3514 & -6.4366 & -4.2600 & 2.3087 & 4.7381] \end{array}$, *H* is hardness, *Fr* is feed rate, *S* is spindle speed, *D* is tool diameter, *L_V_* is viscosity of the lubricant, *C* is the intercept, and *Coeff* is a coefficient.

Many different validation metrics are used for assessing and measuring the agreement between a predictive model and physical observations with the aim of selecting the best models or equations, and choosing the proper validation metric can be a crucial point and a challenge for evaluating results. In this study, the equations developed were compared and validated with the validation metrics listed in Table 9. In order to check the equations in question, different validation metrics were used to test performance on the basis of the results of the equations used in the hardness calculation. The criteria of validation consist in minimising error. To this end, Root Mean Square Error (RMSE) and Mean Absolute Error (MAE) were used for validation in this study. RMSE can be more sensitive to the error in case the MAE is more stable. However, RMSE and MAE are more accurate evaluation metrics compared to other metrics [57]. The equation’s more reliable performance is guaranteed by a condition where MAE and RMSE values are close to 0. Nevertheless, the large variance between RMSE and MAE values represents significant variations in error distribution. Consequently, Mean Relative Error (MRE) was used to measure the precision of the equations applied.

**Figure 10:**
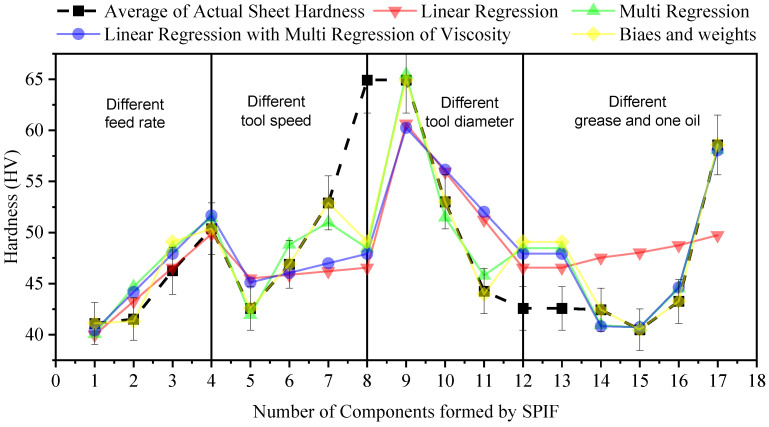
Calculated and real hardness of SPIF components.

As can be seen in Table 9, the suggested Equation Based on Biases and Weight shows much greater reliability compared to other equations, and the next most reliable equation is the Multiple Regression equation. Consequently, both could be applied to precisely calculate the hardness of the SPIF component. Figure 10 illustrates the ability of the developed equations to precisely calculate the hardness of SPIF components compared to the real values of hardness. The fluctuating and uneven hardness in Figure 10 is normal because the values are for different components formed in various conditions using SPIF. All of the hardness data were used in predictions models; by sorting these data from low to high or vice versa will affect the random selection of data as training and testing values, which may make it challenging to distinguish the difference between the actual values of hardness and the predicted values by various models in the figure. The HV of points 1–4 is related to the feed rate, 5–8 is related to the spindle speed, 9–12 is related to the tool diameter, and the rest is related to the four types of grease and one coolant oil. The significant change in the values is because these values were obtained from different components formed in various process conditions. The goal is to employ all of these data to derive different equations despite fluctuations in the data because the mentioned parameter will have parametric values that can be changed in the equations; in the end, the best equation is selected based on the predictive values of hardness that are closest to the actual values with minimum error.

**Figure 11:**
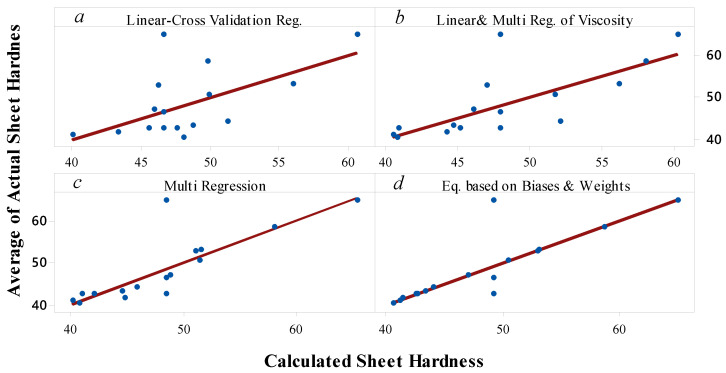
Actual and calculated values obtained with equations of (**a**) Linear Cross-Validation Regression, (**b**) Linear Cross-Validation with Multiple Regression of Viscosity, (**c**) Multiple Regression, and (**d**) Equation Based on Biases and Weights.

The outcomes achieved via the proposed equations versus the real data are presented in Figure 11a–d. The solid line shows a hypothetical exact fit of actual and calculated hardness values, over which data are superimposed. Data dispersion and deviation are based on the ability of the selected equation to predict the hardness values with minimum errors; i.e., a large number of data points that do not match the approximate line (superimposed line) means high error, and a large number of points that match this line means lower error. A satisfactory agreement between the experimental and calculated values was observed for two equations, represented in Figure 11c and d. This figure shows that the equations can appropriately estimate hardness. The connection between the calculated and real data reveals that the calculated values were in agreement with values from the real experiment.

### 3.6. Contribution Analysis of Input Variables

Various methods can be used to evaluate the contribution of each parameter individually and its effective rate on the output. To generate dependent variables, different algorithms for determining the RI of different parameters were employed in this study as a predictor of the predicted output, which is hardness. The algorithms used are Garson’s algorithm [58] and the Most-Squares (MS) algorithm, which was proposed by Ibrahim [59]. The equations of the mentioned algorithms are defined in Equations (6) and (7). Both of the algorithms are based on the connection weights of the neurons of the ANN model, which was built for this purpose using MATLAB R2020a [60]. The network structures consist of input, hidden, and output layers, and the number of neurons is (4-10-1). As a mathematical tool for making predictions in machine learning for the purpose of training multilayer networks, a backpropagation learning algorithm was used: this algorithm is called “multilayer perceptron” (MLP), the concept of which was established by Werbos in 1974 and Rumelhart, McClelland, and Hinton in 1986 [61]. The Garson method has also been used in many studies, as presented in [62,63,64,65,66,67]. Goh [68] applied the Garson algorithm and claimed that RI estimation requires the partitioning of the hidden output weights into elements connected to each neuron in the input layers. Nevertheless, a comparative study of seven different algorithms included the above-mentioned methods to assess relative importance, as outlined in [59]. The author asserted that the Most-Squares method is a better method in comparison to the other methods, and the Most-Squares method seems to outperform all the other methods, as described by the equations below.

(6) $$RI\left(\%\right)=\frac{\left[\sum_{j=1}^{n_{h}}\;(y_{vj\;}/\;\sum_{k=1}^{n_{v}}\;y_{kj})\;hO_{j}\right]}{\sum_{y=1}^{n_{v}}\;\left[\sum_{j=1}^{n_{h}}\;(y_{vj\;}/\;\sum_{k=1}^{n_{v}}\;y_{kj})\;hO_{j}\right]}$$

(7) $$RI\left(\%\right)=\frac{\sum_{j=1}^{n_{v}}{\left(y_{vj}^{i}-y_{vj}^{f}\right)}^{2}}{\sum_{j=1}^{n_{v}}\sum_{v=1}^{n}{\left(y_{vj}^{i}-y_{vj}^{f}\right)}^{2}}$$

where *n_v_* is the number of neurons in the input layer, *n_h_* is the number of neurons in the hidden layer, *y_j_* is the absolute value of connection weights between the input and the hidden layers, *hO_j_* is the absolute value of connection weights between the hidden and the output layers, $\sum_{j=1}^{n_{v}}{\left(y_{vj}^{i}-y_{vj}^{f}\right)}^{2}$ is the sum squared difference between initial connection weights and final connection weights from the input layer to the hidden layer, and $\sum_{j=1}^{n_{v}}\sum_{v=1}^{n}{\left(y_{vj}^{i}-y_{vj}^{f}\right)}^{2}$ is the total of the sum squared difference of all inputs.

The replacement of the input parameters with greater RI significantly influences the outcomes as compared to changes in the parameters with lower RI values [66,67,69]. In the current study, the input parameters are the feed rate, spindle speed, tool diameter and different coolant oils, whereas the outcome is the hardness of the component formed using SPIF.

**Figure 12:**
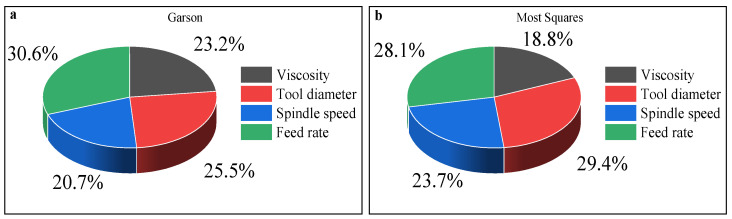
Relative importance of different input variables according to the (**a**) Garson and (**b**) Most-Squares algorithms.

With regard to the relative importance and weights analysis, the most significant factor affecting hardness, as described in Figure 12a, is the feed rate with an RI of 31%, followed by tool diameter with an RI of 25%. Another interesting observation, based on Figure 12b, is a slight difference in the RI ratios. The tool diameter with an RI of 29% is the most influential factor on hardness, followed by feed rate with an RI of 28%. Relying on the Garson method to assess the contribution, it can be claimed that spindle speed and viscosity have nearly the same RI, with values of 23% and 21%, respectively. When the MS method was used, the relative importance of spindle speed and viscosity was 24% and 19%, respectively, the values of which express the effects on hardness. Based on the above-mentioned facts, it can be asserted that the most significant parameters that jointly influence SPIF hardness are the feed rate and tool diameter. A slight difference is acceptable due to the difference in algorithms of the two methods and thanks to the small amount of data studied. It is worth mentioning that the Garson method partitions hidden output connection weights into components associated with each input neuron and uses absolute values of connection weights. At the same time, connection weights between the hidden and the output layers were not used in the MS method: instead, connection weights between the input and hidden layers were used.

## 4. Conclusions

In this study, the effects of four process parameters on hardness were investigated experimentally: the influences of feed rate, spindle speed, and tool diameter on hardness were successfully analysed. Regression equations were established using both linear and quadratic functions based on biases and weights generated from the ANN model of influential forming parameters. Two different methods of RI were used to assess the effects of SPIF parameters on outputs. The RI values of the Garson and Most-Squares methods revealed that feed rate and tool diameter are influential factors, impacting hardness by 29.5% and 27% on average, respectively. Based on our study, the effects of four parameters on hardness can be summarised as follows:

- An increase in the feed rate increases hardness when coolant oil is used. Hardness decreases when grease is used (which happens by way of filling the grooves between asperities with debris carried by the grease).
- The hardness of the component increases when tool speed increases.
- Increases in tool diameter result in a decrease in the hardness of components.
- Grease properties are certain to affect hardness values.
- The use of grease instead of coolant oil generates homogeneous hardness values at different points of the same formed sheet.

From the first four findings, it can be concluded that in order to increase the hardness of a SPIF component made of AA1100 aluminium alloy, high feed rates and high tool speed have to be applied, and coolant oil must be used instead of grease if tools smaller than 8 mm in diameter are used. A significant finding of the study is that the Biases and Weights-Based model, jointly used with Multiple Regression, yielded the best calculation of hardness. In the scope of the calculations, the results were assessed using different validation metrics; in the case of the above-mentioned models, the lowest MRE values were 0.0367 and 0.0555, respectively.

## 5. Recommendations for Further Research

This research presented many issues that require further investigation. Given this, it is worth researching the impact of the studied parameters on sheets of different materials and thicknesses. It is essential to understand whether the mechanical properties of the forming tool affect the hardness of the components or not. A detailed study and evaluation of the different microstructures derived from the different processing conditions of the ISF process should be investigated to examine the impact of the parameters on the grain size, which affects the component hardness. In addition, based on the authors’ knowledge, tool shape and tool material are vital parameters, and the hardness of formed components might be closely related to these parameters, which also merits investigation. Furthermore, different cases should be analysed with different parameter values to test and validate the developed regression equations.

## Figures and Tables

**Table 1:** Mechanical properties of the AA1100 aluminium alloy sheet.

Property	Ultimate Tensile Stress, MPa	Yield Strength, MPa	Elongation, %
Actual	110	95	20
Nominal	110	103	25
Standard Deviation, σ	0	4	2.5

**Table 2:** Chemical composition of the AA1100 aluminium alloy sheet (in wt.%).

Element	Si	Fe	Cu	Mn	Mg	Cr	Ni	Zn	Ti	Pb	B	Sn	V	Al
Actual	0.110	0.482	0.004	0.005	0.001	0.0005	0.004	0.021	0.021	0.0005	0.003	0.001	0.014	balance
Nominal	0.5	0.5	0.2	0.04	0.01	Other 0.15 max								balance

**Table 3:** Selected properties of the greases used.

Grease Type	ISO Viscosity Grade	Average Dropping Point, °C (at 25 °C)	Flash Point, °C	Viscosity at 40 °C, mm^2^/s
EP2	ISO VG 15	90	180	15
Kaucuklu	ISO VG 22	88	172	22
Zinol	ISO VG 32	88	170	32
Gp Grease Calcium	ISO VG 46	58	60	46

**Table 4:** Selected properties of the coolant oil used.

Acidity, pH	Kinematic Viscosity at 29 °C, mm^2^/s	Boiling Point, °C
1.086	1.086	95

**Table 5:** Effects of different feed rates on hardness.

Feed Rate, mm/min	Hardness HV							
	Coolant Oil				Grease			
	Top	Middle	Bottom	Standard Deviation, σ	Top	Middle	Bottom	Standard Deviation, σ
200	43.89	40.40	39.02	2.0494	63.30	58.61	50.56	5.2610
400	39.16	43.61	41.78	1.8262	59.80	56.70	49.13	4.4816
600	44.08	46.44	48.22	1.6957	51.29	53.17	44.70	3.6317
800	47.76	53.84	49.54	2.5522	43.99	45.72	45.50	0.7689

**Table 6:** Effect of different speed values on hardness.

Tool Speed, rpm	Hardness HV			
	Top	Middle	Bottom	Standard Deviation, σ
500	45.83	41.78	40.10	2.4050
1000	50.50	47.48	42.75	3.1895
1500	56.83	55.96	45.90	4.9601
2000	61.58	75.51	57.71	7.6439

**Table 7:** Effects of different tool diameters on hardness.

Tool Diameter, mm	Hardness HV			
	Top	Middle	Bottom	Standard Deviation, σ
4	75.51	57.71	66.61	7.2668
6	56.33	50.65	53.01	2.3299
8	43.22	40.62	44.26	1.5308
10	41.78	40.10	42.57	1.0300

**Table 8:** Effects of different grease types on hardness.

Grease Type	ISO Viscosity Grade	Average Dropping Point (at 25 °C)	Flash Point, °C	Hardness HV			
				Top	Middle	Bottom	Standard Deviation, σ
Gp Grease Calcium	ISO VG 15	58	60	46.81	72.00	56.95	10.3487
Zinol	ISO VG 22	88	170	46.34	42.70	40.74	2.3202
Kaucuklu	ISO VG 32	88	172	42.73	38.12	40.62	1.8843
EP2	ISO VG 46	90	180	45.81	43.30	38.18	3.1751

**Table 9:** Assessment of best alternative equations with different validation metrics for hardness calculation.

Validation Metric	Linear Cross-Validation Regression	Linear Cross-Validation with Multiple Regression of Viscosity	Multiple Regression	Equation Based on Biases and Weights
Mean Error	0.0000	0.0000	−0.0002	−0.0306
Mean Absolute Error	4.8183	3.6826	2.7811	1.8954
Mean Square Error	40.9419	29.2784	22.0436	20.2431
Root Mean Square Error	6.3986	5.4110	4.6951	4.4992
Mean Relative Error	0.0963	0.0727	0.0555	0.0367
Standard Deviation, σ	6.5955	5.5775	4.8396	4.6376
Standard Error of Mean	1.5996	1.3527	1.1738	1.1248

## Data Availability

The data presented in this study are available on request from the corresponding author.
